# Genome-Wide Characterization of Menin-Dependent H3K4me3 Reveals a Specific Role for Menin in the Regulation of Genes Implicated in MEN1-Like Tumors

**DOI:** 10.1371/journal.pone.0037952

**Published:** 2012-05-30

**Authors:** Sunita K. Agarwal, Raja Jothi

**Affiliations:** 1 Metabolic Diseases Branch, National Institute of Diabetes and Digestive and Kidney Diseases, National Institutes of Health, Bethesda, Maryland, United States of America; 2 Systems Biology Section, Biostatistics Branch, National Institute of Environmental Health Sciences, National Institutes of Health, Research Triangle Park, North Carolina, United States of America; Harvard Medical School, United States of America

## Abstract

Inactivating mutations in the *MEN1* gene predisposing to the multiple endocrine neoplasia type 1 (MEN1) syndrome can also cause sporadic pancreatic endocrine tumors. *MEN1* encodes menin, a subunit of MLL1/MLL2-containing histone methyltransferase complexes that trimethylate histone H3 at lysine 4 (H3K4me3). The importance of menin-dependent H3K4me3 in normal and transformed pancreatic endocrine cells is unclear. To study the role of menin-dependent H3K4me3, we performed *in vitro* differentiation of wild-type as well as menin-null mouse embryonic stem cells (mESCs) into pancreatic islet-like endocrine cells (PILECs). Gene expression analysis and genome-wide H3K4me3 ChIP-Seq profiling in wild-type and menin-null mESCs and PILECs revealed menin-dependent H3K4me3 at the imprinted *Dlk1-Meg3* locus in mESCs, and all four *Hox* loci in differentiated PILECs. Specific and significant loss of H3K4me3 and gene expression was observed for genes within the imprinted *Dlk1-Meg3* locus in menin-null mESCs and the *Hox* loci in menin-null PILECs. Given that the reduced expression of genes within the *DLK1-MEG3* locus and the *HOX* loci is associated with MEN1-like sporadic tumors, our data suggests a possible role for menin-dependent H3K4me3 at these genes in the initiation and progression of sporadic pancreatic endocrine tumors. Furthermore, our investigation also demonstrates that menin-null mESCs can be differentiated *in vitro* into islet-like endocrine cells, underscoring the utility of menin-null mESC-derived specialized cell types for genome-wide high-throughput studies.

## Introduction

Whole exome sequencing of different tumor types has identified mutations in various genes whose products are associated with epigenetic processes that are involved in chromatin modification [1]. Sporadic pancreatic endocrine/neuroendocrine tumors of the hormone secreting islet cells of the pancreas harbor inactivating mutations in *MEN1* encoding menin, a component of histone methyltransferase complexes, in 27–44% of tumors [2], [3]. Also, 14–25% of these tumors have mutations in *DAXX* or *ATRX* that encode subunits of a chromatin-remodeling complex [3]. Menin is found in a subset of COMPASS-like (complex of proteins associated with Set1) mixed lineage leukemia (MLL) complexes that trimethylate histone H3 at lysine 4 (H3K4), specifically in MLL1/MLL2-containing complexes that trimethylate H3K4 [4], [5]. The MLL core complex consists of homologs of proteins found in the yeast Set1 histone methyltransferase (HMT) complex such as ASH2, RBBP5, and WDR5.

Menin acts as a tumor suppressor in the autosomal dominant multiple endocrine neoplasia type 1 (MEN1) syndrome characterized by tumors of hormone producing cells of the parathyroids, enteropancreatic endocrine tissues, and anterior pituitary [6]. Menin is essential for early development as indicated by the embryonic lethality at E11.5-E13.5 of homozygous *Men1*-knockout (*Men1*-ko) mouse embryos [7]. *Men1* loss in mouse models driven by RIP-Cre, GLU-Cre, or PDX1-Cre show islet endocrine cell-type restricted tumorigenesis, implicating an essential role for menin in islet endocrine cell homeostasis [7], [8], [9], [10].

Surprisingly, menin's association with MLL is pro-oncogenic in MLL-associated leukemia cells. About 50–60 different translocations involving the MLL1 gene are known to cause acute lymphoid and myeloid leukemias with increased expression of specific homeobox (HOX) genes such as *HOXA7*, *HOXA9*, and the HOX cofactor *MEIS1* [11]. Menin binds to the highly conserved N-terminal 44 amino acids of MLL1 or MLL2; hence, N-terminal MLL peptides could serve as dominant negative inhibitors of the MLL-menin interaction, inhibiting the growth of MLL-transformed leukemic cells (containing MLL-AF9 fusion) by downregulating MLL targets including *HOX* genes and *MEIS1* [12].

The direct role of H3K4 trimethylation (H3K4me3) catalyzed by menin-containing MLL complexes in pancreatic islet endocrine cells is unclear, and the functional relevance of H3K4me3 catalyzed by the MLL1 and MLL2 complexes with or without menin in islet cells has not been elucidated. Pancreatic endocrine cells are found in the islets that comprise only ∼1% of the pancreas. Large quantities of live cells are generally required for most genome-wide occupancy analysis assays. Menin-null human islets are rarely available, which makes islets from MEN1 mouse models (normal and tumor) an attractive alternative source. However, to obtain large quantities of live cells, one would need to isolate and pool islets from a large number of mice. Embryonic stem cells (ESCs) from *Men1*-ko mouse embryos have been established. ESCs can undergo multi-lineage differentiation *in vitro* producing specialized cell types retaining an intact normal diploid karyotype (unlike cell lines and tumors that are aneuploid) [13]. *Men1*-ko (menin-null) mouse ESCs (mESCs) cannot complete hematopoietic differentiation *in vitro* due to reduced *Hoxa9* expression [14]. However, it was not known whether menin-null mESCs could undergo differentiation into pancreatic islet-like endocrine cells *in vitro*.

**Figure 1:**
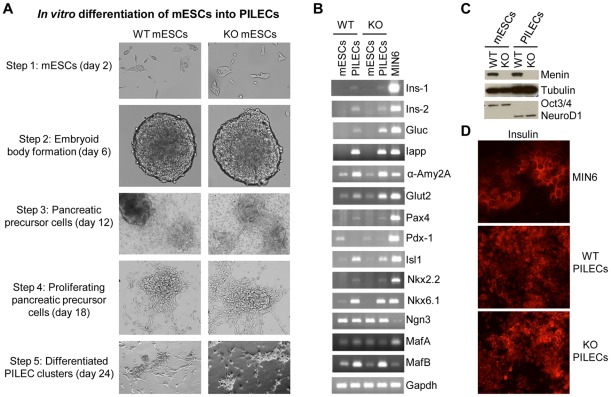
*In vitro* differentiation of mESCs into pancreatic islet-like endocrine cells (PILECs). (A) Phase contrast images of wild-type (WT) mESCs and menin-null (KO) mESCs differentiated into PILECs. (B) RT-PCR analysis measuring mRNA levels of genes expressed in islet cells using RNA from mESCs and mESC-derived PILECs. WT: wild-type cells; KO: *Men1*-ko cells (menin-null). RNA from the mouse insulinoma cell line MIN6 was used as a positive control. Gapdh served as an internal control for RT-PCR using a 1∶10 dilution of the oligo-dT primed first strand cDNA template. (C) Western blot analysis of whole cell protein extracts from wt or menin-null mESCs before and after differentiation into PILECs with antibodies against menin, an ESC pluripotency marker Oct3/4, and an islet differentiation marker NeuroD1. Tubulin served as protein loading control. (D) *In vitro* differentiation of pancreatic precursor cells (step-3) derived from mESCs into PILECS was performed in gelatinized chamber slides and processed for immunofluorescence (red) staining with a pro-insulin C-peptide mouse monoclonal antibody to detect insulin. MIN6 cells cultured in chamber slides were used as a positive control.

We performed *in vitro* differentiation of wild-type as well as menin-null mESCs into pancreatic islet-like endocrine cells (PILECs) in order to obtain a source of cells with a homogenous and diploid genetic background for global menin-dependent H3K4me3 and gene expression analyses. We used ChIP-Seq and microarray analysis to profile genome-wide H3K4me3 and gene expression, respectively, in wild-type and menin-null mESCs and PILECs. Specific and significant loss of menin-dependent H3K4me3 was observed at imprinted *Dlk1-Meg3* locus in menin-null mESCs, and at all four *Hox* loci in menin-null PILECs. These H3K4me3 losses were accompanied by reductions in gene expression. Meg3 (maternally expressed gene 3) is an imprinted long non-coding RNA that acts as a tumor suppressor [15]. Given that the reduced expression of genes at the *DLK1-MEG3* and *HOX* loci is associated with sporadic pituitary tumors and parathyroid tumors, respectively [16], [17] (endocrine tumor types also found in the MEN1 syndrome), our data suggests a possible role for menin-dependent H3K4me3 at these genes in the initiation and progression of sporadic pancreatic neuroendocrine tumors.

**Figure 2:**
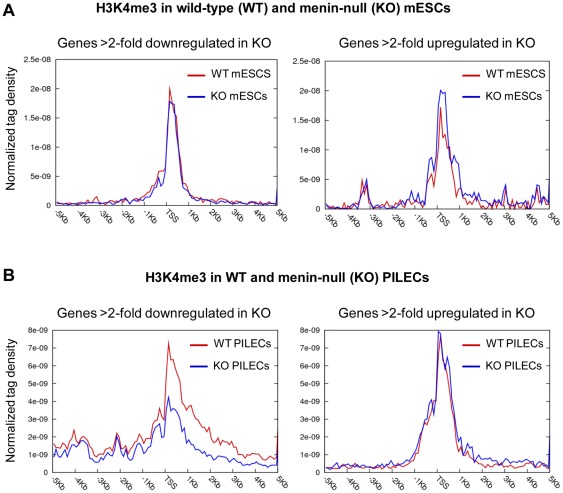
Decrease in gene expression accompanies decrease in H3K4me3 in menin-null PILECs but not in menin-null mESCs. Correlation between changes in gene expression and changes in H3K4me3 in menin-null (KO) mESCs vs. wild-type (WT) mESCs (A), and menin-null PILECs vs. wild-type (WT) PILECs (B). Normalized average tag density surrounding the transcription start site (TSS) is shown for genes that were at least 2-fold downregulated/upregulated in menin-null cells compared to WT cells.

## Results

### Menin-null mESCs can differentiate into pancreatic islet-like endocrine cells *in vitro*

*Men1*-ko (menin-null) mouse embryonic stem cells (mESCs) were deficient in completing hematopoietic differentiation *in vitro* [14]. In order to determine whether loss of menin affects the development of islet endocrine cells, we performed *in vitro* differentiation of menin-null mESCs into pancreatic islet-like endocrine cells (PILECs). Menin-null mESCs used in our study showed no growth defects, and were not compromised for embryoid body (EB) formation (**Figure** **1A**) which is consistent with previous reports [14], [18], [19]. Morphological changes during the differentiation of wild-type (wt) and menin-null cells into PILECs were similar (**Figure** **1A**). *In vitro* differentiation of both wt and menin-null mESCs into PILECs was assessed by measuring the expression of marker transcripts characteristic of islet cells. We observed comparable levels of the markers in both wt and menin-null mESC-derived PILECs (**Figure** **1B**). In menin-null mouse models, either the development of islets was unaffected or the development of early pancreatic endocrine cells was impaired [18], [20]. Our data show that menin is not essential for the development of islet-like endocrine cells.

**Figure 3:**
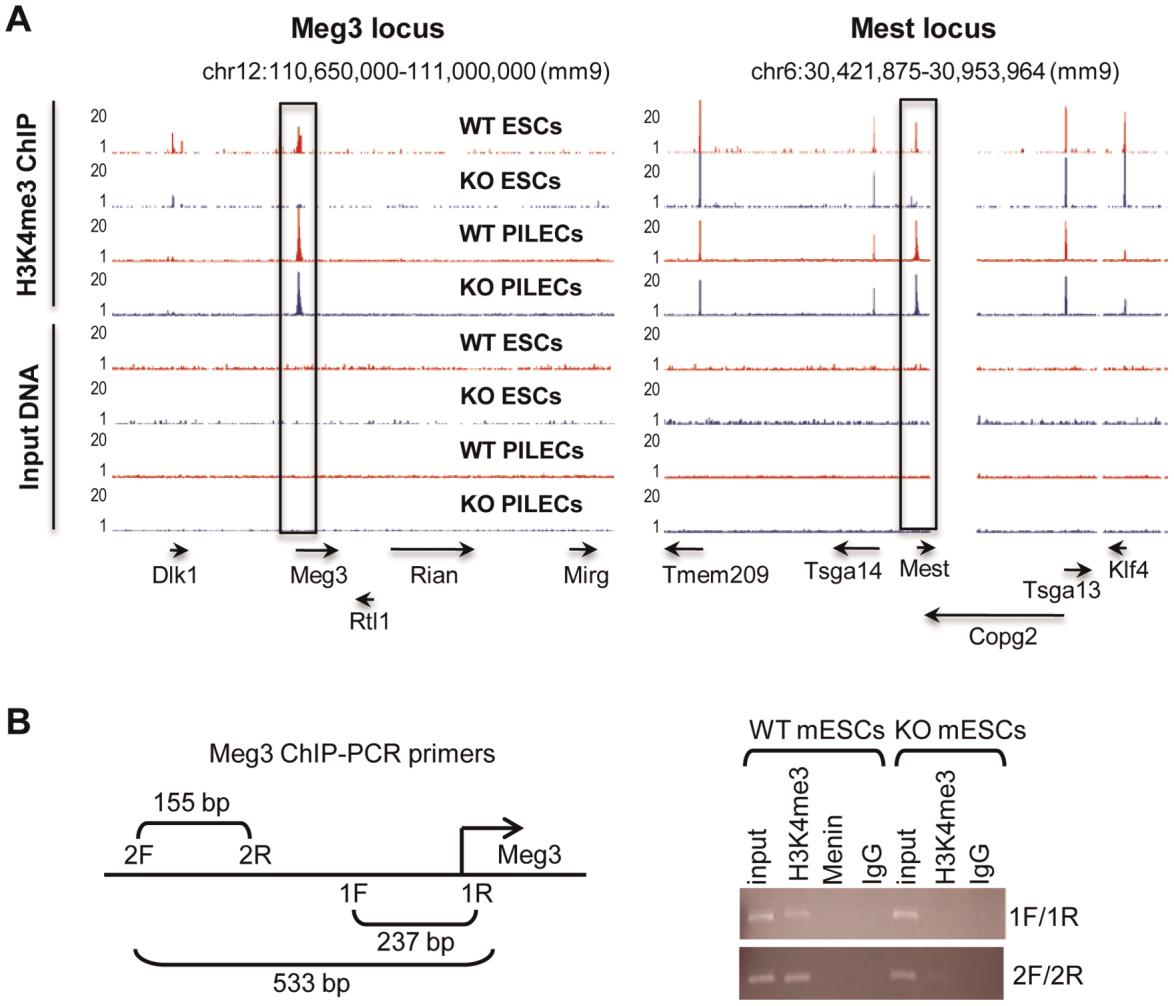
H3K4me3 at the *Meg3* promoter is menin-dependent in mESCs, but not in PILECs. (A) UCSC genome browser images of H3K4me3 profiles (top four tracks) at *Meg3* (left) and *Mest* (right) loci in wild-type (WT) and menin-null (KO) mESCs and PILECs. The bottom four tracks show profiles for control Input DNA. Rectangular box highlights the promoter regions of *Meg3* and *Mest*. Genes within the two loci and their orientation are marked using arrows at the bottom. (B) Schematic (left) showing the *Meg3* promoter region and the location of the ChIP-PCR primers and product length. Transcriptional start site is marked with the forward arrow. Results from ChIP-PCR analysis (right) from control input DNA, and DNA from ChIPs with antibodies against H3K4me3, menin, and normal rabbit IgG.

**Figure 4:**
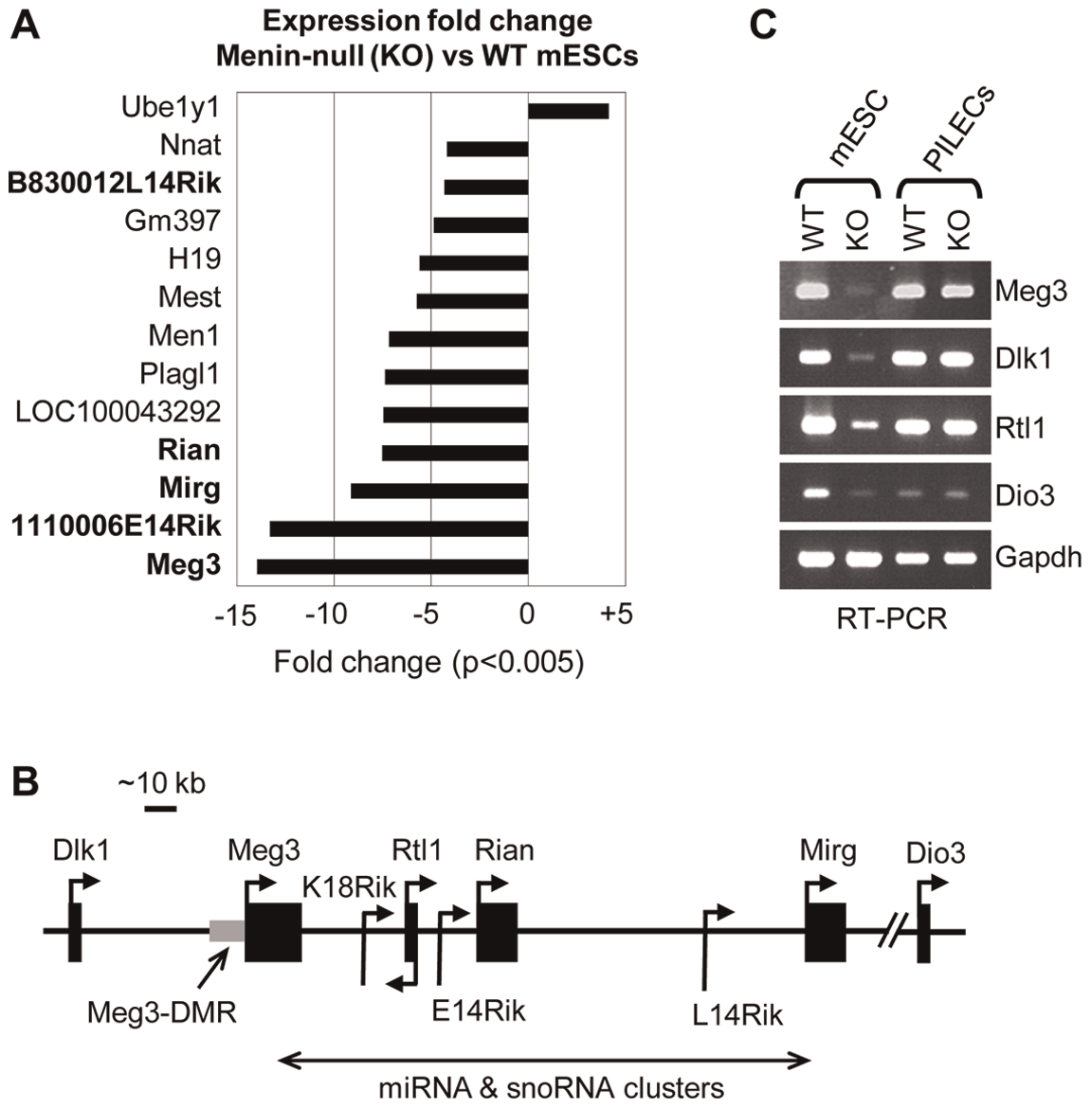
Menin-dependent regulation of genes within the *Dlk1*-*Meg3* locus in mESCs, but not in PILECs (A) Expression fold changes of genes that showed at least 4-fold change (p-value <0.005) in menin-null (KO) mESCs vs wild-type (WT) mESCs in microarray analysis. Genes marked in bold are from the *Dlk1-Meg3* locus. (B) Schematic showing the *Dlk1*-*Meg3* locus of ∼300 kb region near *Meg3* on chromosome 12 showing the relative location of the nearby genes and their orientation of expression indicated with arrows, and the location of noncoding RNAs (miRNAs and snoRNAs). (C) RT-PCR analysis measuring mRNA levels of Meg3 and other coordinately regulated genes (Dlk1, Rtl1 and Dio3) within the *Dlk1-Meg3* locus using RNA from WT and KO mESCs and PILECs. Gapdh served as an internal control for RT-PCR using a 1∶10 dilution of the oligo-dT primed first strand cDNA template.

Menin expression levels in mESC-derived PILECs were largely similar to that in wt mESCs. Upon differentiation of both wt and menin-null mESCs, the ESC pluripotency marker Oct4 was similarly downregulated and the differentiation marker NeuroD1 protein was similarly upregulated (**Figure** **1C**). Also, anti-C-peptide antibody immunofluorescence of the PILEC clusters showed similar staining for insulin in wt and menin-null cells (**Figure** **1D**). Whether PILECs are capable of producing and secreting physiological levels of insulin that is regulated by glucose has not been firmly established [21]. However, low-to-moderate level of insulin encoding transcripts (Ins-1 and Ins-2) and other islet hormone encoding transcripts (Gluc and Iapp) were observed in both wt and menin-null cells after differentiation (**Figure** **1B**). Therefore, we concluded that lack of menin did not affect the ability of mESCs to differentiate into the islet endocrine lineage *in vitro*, and that PILECs derived through *in vitro* differentiation of wt or menin-null mESCs could serve as a good surrogate for wt or menin-null islet endocrine cells, respectively.

**Figure 5:**
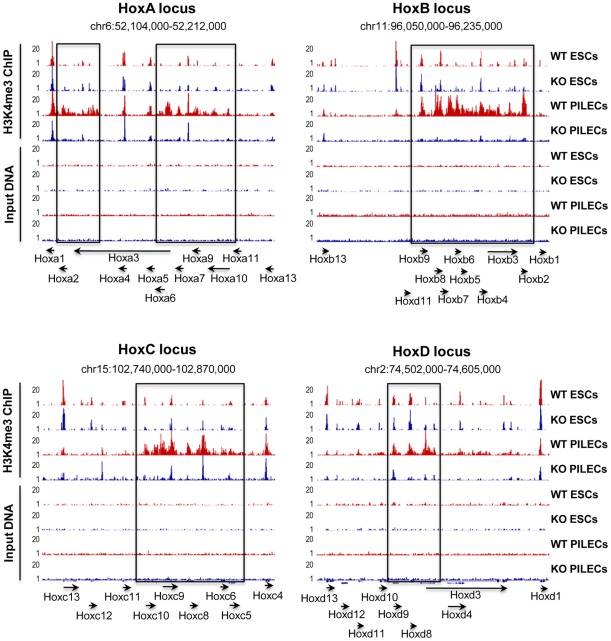
H3K4me3 at the four *Hox* loci is menin-dependent in PILECs UCSC genome browser images of H3K4me3 profiles (top four tracks) at the four *Hox* loci in wild-type (WT) and menin-null (KO) mESCs and PILECs. The bottom four tracks show profiles for control Input DNA. Rectangular box highlights the regions showing differential H3K4me3 in WT and KO PILECs. Genes within the four *Hox* loci and their orientation are marked using arrows at the bottom.

### Genome-wide mapping of H3K4me3 and gene expression profiling in wt and menin-null cells

Histone methyltransferase complexes contain menin, specifically the MLL1- and MLL2-containing COMPASS-like MLL complexes that are known to deposit the histone H3 lysine 4 trimethyl mark (H3K4me3). Homozygous *Men1*-ko mice are early embryonic lethal, and the role of menin-dependent H3K4me3 in embryonic lethality is unknown. To examine menin-dependent H3K4me3, we used ChIP-Seq to perform genome-wide mapping of regions enriched for H3K4me3 in wt and menin-null mESCs and PILECs. To ensure that the data we generated is of high quality, we compared our H3K4me3 data from wt mESCs with a previously published H3K4me3 data from the same cell type [22], and found that they were highly similar in specificity and sensitivity (**Figure** **S1A**). H3K4me3 marks were highly enriched within the genic regions of the genome. In particular, H3K4me3 marks were specifically observed at proximal promoter regions near transcriptional start sites (TSSs) (**Figure** **S1B**).

**Figure 6:**
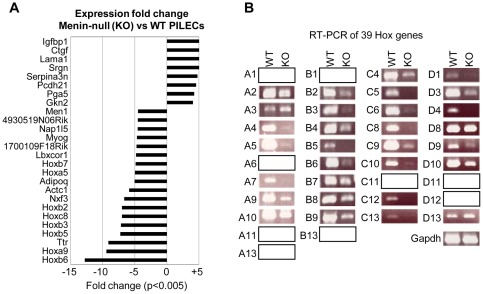
Menin-dependent regulation of *Hox* genes in PILECs. (A) Expression fold changes of genes that showed at least 4-fold change (p-value <0.005) in menin-null (KO) PILECs vs wild-type (WT) PILECs in microarray analysis. (B) RT-PCR analysis measuring mRNA levels of 39 *Hox* genes from the 4 *Hox* clusters (*HoxA*, *HoxB*, *HoxC* and *HoxD*) using RNA from wild-type (WT) or menin-null (KO) mESC-derived PILECs. Gapdh served as an internal control for RT-PCR using a 1∶10 dilution of the oligo-dT primed first strand cDNA template. Blank boxes represent *Hox* genes whose expression was undetectable in the WT or KO cells.

To determine the link between menin-dependent H3K4me3 and transcription, we used microarrays to profile global gene expression in wt and menin-null mESCs and PILECs. Seventy one and 167 genes were at least 2-fold differentially expressed (p-value < 0.005) in menin-null mESCs and menin-null PILECs, respectively (**Figure** **S2**, and **Tables** **S1** and **S2**). Examination of H3K4me3 levels at genes that were at least 2-fold upregulated or 2-fold downregulated in menin-null mESCs compared to wt mESCs revealed that expression changes in menin-null vs wt mESCs cells did not accompany changes in H3K4me3 levels (**Figure** **2A**). However, we found that genes that were at least 2-fold downregulated in menin-null PILECs underwent significant reduction in H3K4me3 levels (**Figure** **2B**). These data suggested a direct role for menin-dependent H3K4me3 in the regulation of genes in PILECs, and a rather limited and indirect role in mESCs.

**Figure 7:**
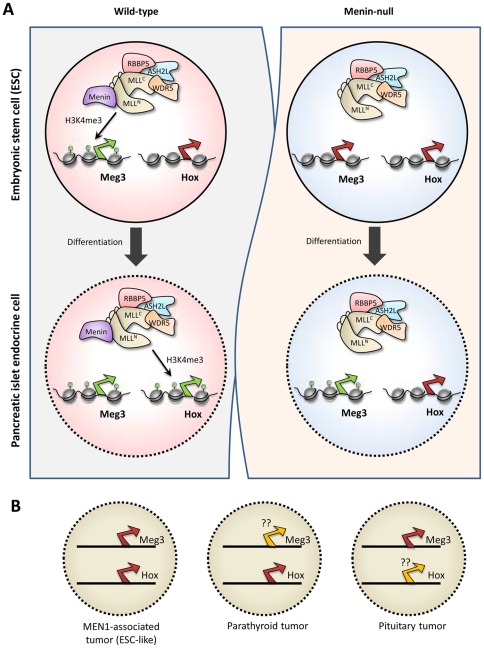
Proposed model for menin-dependent H3K4me3-mediated regulation of gene expression. (A) Left and right panels show gene expression status of genes regulated by menin-dependent H3K4me3 as the wild-type and menin-null embryonic stem cells (ESCs), respectively, differentiate into pancreatic islet-like endocrine cells. H3K4me3 marks along the chromatin is shown using filled green circles. Transcription start sites of *Meg3* and *Hox* genes are marked using a green (expressed) or a red (silent) arrow. Menin positively regulates Meg3 expression in ESCs, but not in islet cells. The loss of menin results in the loss of Meg3 expression in ESCs, but not in islet cells. In contrast, menin positively regulates the expression of Hox genes in islet cells, but not in ESCs. *Hox* genes are silenced in menin-null islet. (B) The gene expression status of *MEG3* and *HOX* genes in MEN1-like tumors types (left), parathyroid tumors (middle), and pituitary tumors (right) as previously reported [16], .

### Menin-null mESCs exhibit loss of H3K4me3 at the *Meg3* promoter

Since the genome-wide H3K4me3 profiles were largely similar between wt and menin-null mESCs (**Figure** **2A**), we systematically began identifying regions with differential H3K4me3. Only a handful of genomic regions showed quantitative loss of H3K4me3 in menin-null mESCs (**Table** **S3**), suggesting that the role of menin-dependent H3K4me3 in mESCs is limited and specific. Since a vast majority of these regions were intergenic, we focused on those that were near genes. Among the most significant differentially H3K4 trimethylated regions between wt and menin-null mESCs were the promoters of imprinted *Meg3* and *Mest* (mesoderm specific transcript) genes that experienced complete loss of H3K4me3 in menin-null mESCs compared to wt mESCs (**Figure** **3A**). Interestingly, no obvious change in H3K4me3 levels at the *Meg3* or *Mest* promoters in menin-null PILECs compared to wt PILECs was observed (**Figure** **3A**). ChIP-PCR assay confirmed the loss of H3K4me3 at the *Meg3* promoter in menin-null mESCs but not in menin-null PILECs (**Figure** **3B**). These data suggested a mESC specific role for menin in regulation of *Meg3* and *Mest*.

### Menin regulates genes within the *Dlk1*-*Meg3* locus in mESCs but not in PILECs

To determine the connection, if any, between the loss of menin-dependent H3K4me3 and transcription, we examined the microarray gene expression data in wt and menin-null mESCs. Since H3K4me3 is generally associated with active and poised gene promoters [22], [23], we hypothesized that the loss of H3K4me3 at the *Meg3* and *Mest* promoters in menin-null mESCs accompanies loss of Meg3 and Mest expression. Examining the list of genes differentially expressed between wt and menin-null mESCs confirmed that this was indeed the case (**Table** **S1**). Meg3 and Mest were expressed 14-fold and 6-fold less (p<0.005) in menin-null mESCs compared to wt mESCs.

Microarray analysis revealed few other genes that had reduced expression in menin-null mESCs but showed no changes in their H3K4me3 levels compared to wt mESCs (**Table** **S1** and **S3**). Among them were five transcripts encoded from genes within the ∼300 Kb *Dlk1-Meg3* locus (1110006E14Rik, Rian, B830012L14Rik, and Mirg) that had ∼4–13 fold reduced expression in menin-null mESCs compared to wt mESCs (**Figure** **4A**). These transcripts along with Meg3, Dlk1, Rtl1, and Dio3 are known to be coordinately regulated and controlled by the Meg3-DMR (differentially methylated region) [24] that overlaps with the *Meg3* promoter region where H3K4me3 was lost in the menin-null mESCs (**Figure** **4B**). RT-PCR assay confirmed the reduced expression of Meg3, Dlk1, Rtl1, Dio3 in menin-null mESCs (**Figure** **4C**). Unlike menin-null mESCs, the differentiated PILECs derived from menin-null mESCs showed no significant differences in the expression of genes within the imprinted *Dlk1-Meg3* locus nor promoter-bound H3K4me3 levels at the *Meg3* promoter overlapping Meg3-DMR (**Figure** **3A** and **4C**). Therefore, we concluded that the positive regulation of genes within the *Dlk1-Meg3* is menin-dependent only in mESCs but not in the differentiated PILECs.

### Menin-null PILECs have reduced H3K4me3 at the *Hox* loci and reduced expression of *Hox* genes

Lack of menin did not affect the ability of mESCs to differentiate into the islet endocrine lineage *in vitro*. However, given that the loss of menin is known to lead to pancreatic neuroendocrine tumors [2], [3], we sought to determine the effect of menin loss in differentiated PILECs. Genome-wide mapping and analyses of H3K4me3 profiles in PILECs derived from wt and menin-null mESCs revealed that among the most significant differentially H3K4 trimethylated regions were the loci containing the four *Hox* clusters (**Figure** **5** and **Table** **S4**). The *Hox* clusters, each containing 9 to 11 genes, are located on chromosomes 6, 11, 15, and 2 in the mouse genome. The 39 *HOX* genes are a highly conserved family of transcription factors essential for body axis patterning, development and differentiation. Although *HOX* genes are essential during embryogenesis, their regulation and target genes are not well defined. *HOX* genes are epigenetically silenced in undifferentiated ESCs, and their activation is associated with differentiation. *Hox* genes in mESCs are known to be marked by bivalent histone modifications of active H3K4me3 and repressive H3K27me3 [22]: H3K27me3 keeps these genes silenced in mESCs, while the H3K4me3 keeps them poised for future activation. We observed low levels of H3K4me3 at the *Hox* loci in both wt and menin-null mESCs (**Figure** **5**). Upon differentiation into PILECs, all four *Hox* loci in wt cells exhibited a significant increase in H3K4me3 levels, whereas such increase was not evident in differentiated menin-null cells. Interestingly, the increase in H3K4me3 at the *Hox* loci in wt PILECs was in the form of broader H3K4me3 footprints extending well past the proximal promoter regions and into the gene bodies (**Figure** **5**). Given that it was previously shown using ChIP-chip that menin colocalizes with H3K4me3 at the *Hox* loci [25], we concluded that the broad H3K4me3 footprints in wt PILECs were clearly menin-dependent.

Gene expression microarray analysis revealed that the muted levels of H3K4me3 at *Hox* loci in menin-null PILECs were associated with significant and proportional reduction in the expression of many *Hox* genes compared to wt PILECs (**Figure** **6A** and **Table** **S2**). RT-PCR assay confirmed the loss (or several-fold reduction) of expression for many *Hox* genes (**Figure** **6B**). Together, these data indicated that menin is required for the expression of *Hox* genes in pancreatic islet cells, and that its role in the regulation of *Hox* genes is probably via its H3K4me3-associated activity as a member of MLL1/MLL2 containing MLL histone methyltransferase complex.

## Discussion

Using genomic data generated from ChIP-Seq and gene expression microarrays in wt and menin-null mESCs and mESC-derived pancreatic islet-like endocrine cells (PILECs), we found that menin loss results in a significant loss of H3K4me3 only at a limited number of loci in mESCs and PILECs, thus defining a specific role for menin in modulating gene expression at these loci (**Figure** **7A**). We identified the *Dlk1*-*Meg3* locus in mESCs, and the four *Hox* loci in PILECs to be regulated by menin in an H3K4me3-dependent manner. This is consistent with a previous ChIP-chip study in human islets that showed colocalization of menin with H3K4me3 at the *Hox* loci [25], and another ChIP-chip study that showed loss of H3K4me3 at the *Hox* clusters in menin-null mouse embryonic fibroblasts [26]. How menin gets recruited to the *MEG3* locus remains to be determined. The *MEG3* and *HOX* loci, identified in this study to be regulated by menin-dependent H3K4me3, assumes significance given that genes within these loci have been implicated in MEN1-like tumors types: silencing of *MEG3* in pituitary tumors and *HOX* genes in parathyroid tumors [16], [17] (**Figure** **7B**).

The *Dlk1-Meg3* region on mouse chromosome 12 (human chromosome 14q32) is an imprinted locus consisting of multiple maternally expressed noncoding RNA genes and paternally expressed protein-coding genes (**Figure** **4C**). *MEG3* encodes a noncoding RNA that acts as an imprinted tumor suppressor gene. Reduced *MEG3* expression and promoter DNA hypermethylation has been observed in various human tumor types: pituitary adenomas, neuroblastomas, pheochromocytomas, Wilms tumors, and other carcinomas [27]. Furthermore, most of the genes at the *DLK1-MEG3* locus are selectively silenced in clinically nonfunctioning pituitary adenomas, ACTH-secreting pituitary adenomas and PRL-secreting pituitary adenomas [16]. Somatic *MEN1* mutations are observed in 30% of the common sporadic counterparts (e.g., parathyroid adenoma, gastrinoma, insulinoma and bronchial carcinoid) of the endocrine tumor types seen in familial MEN1, except in sporadic pituitary tumors (only 1–5% with *MEN1* mutation) [28]. Identification of *Meg3* as a menin target gene provides insights into the role of menin-regulated genes as potential candidates for pituitary tumorigenesis. *Meg3* is essential for embryonic development indicated by the premature lethality of *Meg3* knockout mice; therefore, the role of *Meg3* in tumor development could not be assessed in mouse models [29], [30]. Early developmental factors are targets of mutation or aberrant expression in cancers whereby tumors acquire ESC-like self-renewal properties. Although *Meg3* silencing did not affect the viability or proliferation of menin-null mESCs, its silencing in mature differentiated cells *in vivo* upon menin loss or from other causes could contribute to tumorigenesis. Our data suggests that perhaps other menin-independent mechanisms, activated during the differentiation of mESCs into PILECs, induce the expression of transcripts from the *Dlk1-Meg3* locus, which would mean that the *Meg3* locus is essential for PILEC differentiation. Identification of such mechanisms could lead to a better understanding of *Meg3*'s role in early differentiation and in tumorigenesis.

*HOX* gene expression in mature adult cells is essential for the maintenance of cellular identity; for instance, in cells with high turnover such as in the proliferation and differentiation of blood cells. Abnormal *HOX* gene expression has been observed not only in acute leukemias but has also been associated with oncogenesis in breast, cervical, lung, ovarian, prostate, and thyroid cancers [31]. For MLL-mediated leukemogenesis, MLL fusion proteins cause constitutive *HOX* gene activation; without menin, specific *HOX* gene expression is reduced thus preventing leukemogenesis [32]. Quantitative RT-PCR analysis of the 39 *HOX* genes by an earlier study showed upregulation of 23 *HOX* genes among familial MEN1 parathyroid tumors with biallelic *MEN1* loss, and downregulation of 5 *HOX* genes among sporadic parathyroid tumors without *MEN1* loss [17]. This is consistent with observations in other tumor types (e.g., breast) where both up- and down-regulation of specific *HOX* genes was noted [31]. Therefore, although menin-dependent *Hox* gene expression is not essential for differentiation of mESCs into islet endocrine lineage *in vitro*, it could be causative for endocrine tumorigenesis in mature cells. However, the role of *HOX* downregulation in pancreatic endocrine tumors from menin loss or from other causes needs to be determined.

Further investigations analyzing the regulation of menin targets (*HOX* and *DLK1-MEG3* genes) during the initiation and progression of tumors found in MEN1 or MEN1-like sporadic endocrine tumors will be instrumental in correlating these events as biomarkers and/or causes of endocrine neoplasia.

Our investigation demonstrates the utility of mESCs differentiated *in vitro* into pancreatic islet-like endocrine cells for genome-wide analysis studies. Also, mESC-derived islet-like cells could be used to examine factors that might help restore H3K4me3 that is lost upon menin deficiency. Several labs are conducting improvements in differentiation protocols for hormone enriched pancreatic endocrine cells for replacement therapy in diabetes and other diseases [21]. Such protocols may help extend our analysis of the genome-wide menin-dependent histone modifications, and may facilitate the *in vitro* analyses of factors that could counteract menin deficiency. Available protocols for *in vitro* differentiation for specific tissues affected by tumors from menin loss such as adipocytes, parathyroids, and anterior pituitary [33], [34], [35] could be used for generating specialized cells for similar genome-wide studies. This will facilitate our understanding of menin-dependent molecular and cellular processes in development and those disrupted in neoplasia.

## Methods

### Antibodies

The following antibodies were used: rabbit anti-H3K4me3 (Upstate, 07–473), rabbit anti-menin (Bethyl, A300–105A), rabbit anti-NeuroD1 (Aviva, ARP32036_T100), rabbit anti-Oct3/4 (Santa Cruz, sc-9081), mouse anti-Tubulin (Calbiochem, CP06), normal rabbit IgG (Santa Cruz, sc-2027), HRP-conjugated rabbit and mouse secondary antibodies (Santa Cruz, sc-2054 and sc-2055).

### mESC culture

Wild-type mESCs (TC-1) and *Men1*-ko (menin-null) mESCs (3.2N) [14] were cultured on a feeder layer of IRR-STO irradiated primary MEFs (ATCC) in ESC maintenance medium containing Leukemia Inhibitory Factor (LIF). The mESCs were rendered feeder-free by several rounds of culturing for short periods of time (30 mins) on tissue culture treated dishes to get rid of the attached feeder layer cells, and the unattached mESCs were ultimately cultured on gelatin-coated dishes.

### Differentiation of mESCs into pancreatic islet-like endocrine cells (PILECs)

Wild-type mESCs and menin-null mESCs were differentiated into pancreatic islet-like endocrine cells (PILECs) [36] with the reagents and protocol, ‘In Vitro Differentiation of Mouse Embryonic Stem Cells into Insulin Secreting Pancreatic Islet-like Clusters’ provided by the manufacturer (STEMCELL Technologies). Briefly, mESCs were plated onto gelatinized dishes in ESC maintenance medium containing LIF for 2 days, followed by embryoid body (EB) formation in ultra-low adherent dishes as suspension culture in the same medium without LIF for 2 days. EBs were plated onto gelatinized dishes in serum-free ITS-A supplemented medium for 6 days. These pancreatic precursor cells were trypsinized and first expanded on gelatinized dishes in pancreatic proliferation medium (serum-free medium with N2 and B27 supplements, and FGF-β) for 6 days; then cells were induced to differentiate in the same dishes into pancreatic islet-like cells in the presence of nicotinamide (in serum-free medium with N2 and B27 supplements, but without FGF-β) for 6 days. Microscopy and photomicrography was performed with the Axio Observer.Z1 inverted microscope (Zeiss). Pancreatic precursor cells were also plated in gelatinized chamber slides for expansion and differentiation into pancreatic islet-like cells. These chamber slides were later used for assessing insulin by immunofluorescence. For chromatin lysate preparation used in ChIP and ChIP-Seq, differentiation was performed using EBs from 2×10^6^ mESCs in each 100 mm dish.

### RNA, gene expression microarray, and RT-PCR

Total RNA was isolated using Trizol (Invitrogen) and further purified using the RNeasy kit (Qiagen) from 2 independent cultures of wt or menin-null mESCs and 2 independent cultures of differentiated wt or menin-null PILECs. RNA quality was assessed on the Agilent Bioanalyzer. Each sample was processed and analyzed at the NIDDK microarray core facility using an Affymetrix microarray platform. Labeled RNA samples were hybridized to Affymetrix Genechip mouse genome 430, 2.0 array. Microarray data were normalized and analyzed using the Affymetrix Genechip software, Microarray Analysis Suite 5.0. For RT-PCR, DNase I (Ambion) treated RNA samples were reverse transcribed using oligo-dT and SuperScript III (Invitrogen), and the first strand cDNA was used for PCR in standard PCR reactions with Taq Gold (Applied Biosystems). PCR products were analyzed by agarose gel electrophoresis. The primers sequences are listed in **Table S5**.

### Western blot

Whole cell protein extract (WCE) from wt or menin-null mESCs, and wt or menin-null mESCs differentiated into PILECs was prepared in buffer containing 1X TBS, 0.1% Igepal, and protease inhibitors (Roche). Protein concentrations were determined with a detergent-compatible protein assay (Bio-Rad). Equal amount of proteins (50 µg) were separated by SDS-PAGE, electro-blotted onto nitrocellulose membranes, and detected with appropriate antibodies and ECL (Millipore).

### Immunofluorescence

mESCs differentiated into PILECs in gelatinized chamber slides were fixed in 4% paraformaldehyde, permeabilized with 0.5% Triton X-100, and stained with pro-insulin C-peptide mouse monoclonal antibody (Millipore, AB1342) and anti-mouse secondary antibody conjugated to Texas Red (Jackson ImmunoResearch Laboratories). Microscopy and photomicrography was performed with an epifluorescence microscope (Zeiss).

### Chromatin immunoprecipitation (ChIP) and ChIP-Seq

Cells in culture dishes were cross-linked with 1% formaldehyde, and processed for chromatin lysate preparation, and chromatin immunoprecipitation (ChIP) using the ChIP assay kit (Millipore). Chromatin lysate was sonicated with a Bioruptor system (Diagenode) to yield a DNA smear averaging 250 bp. Sonicated chromatin lysate from 2×10^6^ mESCs undifferentiated or differentiated was used for each ChIP with 5 µg antibody (H3K4me3, menin, JunD, IgG). About 20 ng of DNA was obtained from each H3K4me3 ChIP of chromatin from 2×10^6^ cells. ChIP DNA was used for ChIP-PCR assays or for preparation of ChIP-Seq libraries. ChIP-PCR was performed in standard reaction conditions with Taq Gold (Applied Biosystems), and the products were analyzed by agarose gel electrophoresis. The primers sequences are listed in Table S5. For ChIP-Seq libraries, 20 ng of input chromatin DNA or ChIP DNA was processed using the ChIP-Seq sample prep kit (Illumina). Gel purified ChIP-Seq library DNA was further purified by phenol-chloroform extraction and ethanol precipitation, and processed for cluster generation, 36 cycle sequencing, and sequence analysis using Illumina GAII.

### ChIP-Seq data analysis

Sequenced 36-bp reads were aligned to the mouse reference genome (mm9 assembly), and only those reads/tags that mapped to unique genomic locations with at most two mismatches were retained for further analysis. The mapped tags for each sample were converted to a browser extensible data (BED) file, detailing the genomic coordinate of each tag. Summary files, displaying the normalized number of tags in 200-bp windows, in BED format were used for viewing in the UCSC Genome Browser, and to generate screenshots. For generating tag density plots, data across samples were normalized by the total number of reads within each sample.

### Data availability

All the ChIP-Seq and Gene expression microarray data generated for this study were deposited in the NCBI GEO repository under the accession number GSE37776.

## Supporting Information

Figure S1 **Data quality of genome-wide H3K4me3 ChIP-Seq from mESCs.** (A) UCSC genome browser images of H3K4me3 profiles (top two tracks) at a randomly selected ∼600 Kb region on chromosome 17 in wild-type mESCs. The top track shows H3K4me3 data from a previously published report (17). The middle and bottom track shows the data (H3K4me3 and input, respectively) generated for this study. Genes within the locus are shown at the bottom. (B) Normalized average tag density across a gene unit (left) and 10 Kb surrounding the transcription start site (TSS) (right) is shown. All genes in the mouse genome were used to calculate the average tag density.
(TIF)

Figure S2 **Differentially expressed genes in menin-null cells.** Number of genes that were at least 2-fold differentially expressed (p<0.005) in menin-null vs wild-type (WT) mESCs and menin-null vs WT pancreatic islet-like endocrine cells (PILECs) are shown. Overlaps between the differentially expressed subsets are represented as venn diagrams.
(TIF)

Table S1 Genes differentially expressed in menin-null vs wild-type mESCs.
(XLSX)

Table S2 Genes differentially expressed in menin-null vs wild-type pancreatic islet-like endocrine cells.
(XLSX)

Table S3 Genomic regions with differential H3K4me3 in menin-null vs wild-type mESCs.
(XLSX)

Table S4 Genomic regions with differential H3K4me3 in menin-null vs wild-type pancreatic islet-like endocrine cells.
(XLSX)

Table S5 PCR Primers.
(XLSX)
