# Supplementary figures and images for: Genome-Wide Characterization of Menin-Dependent H3K4me3 Reveals a Specific Role for Menin in the Regulation of Genes Implicated in MEN1-Like Tumors
Source: PLoS One. 2012 May 30;7(5):e37952. doi: 10.1371/journal.pone.0037952 (PMC3364203; doi:10.1371/journal.pone.0037952)

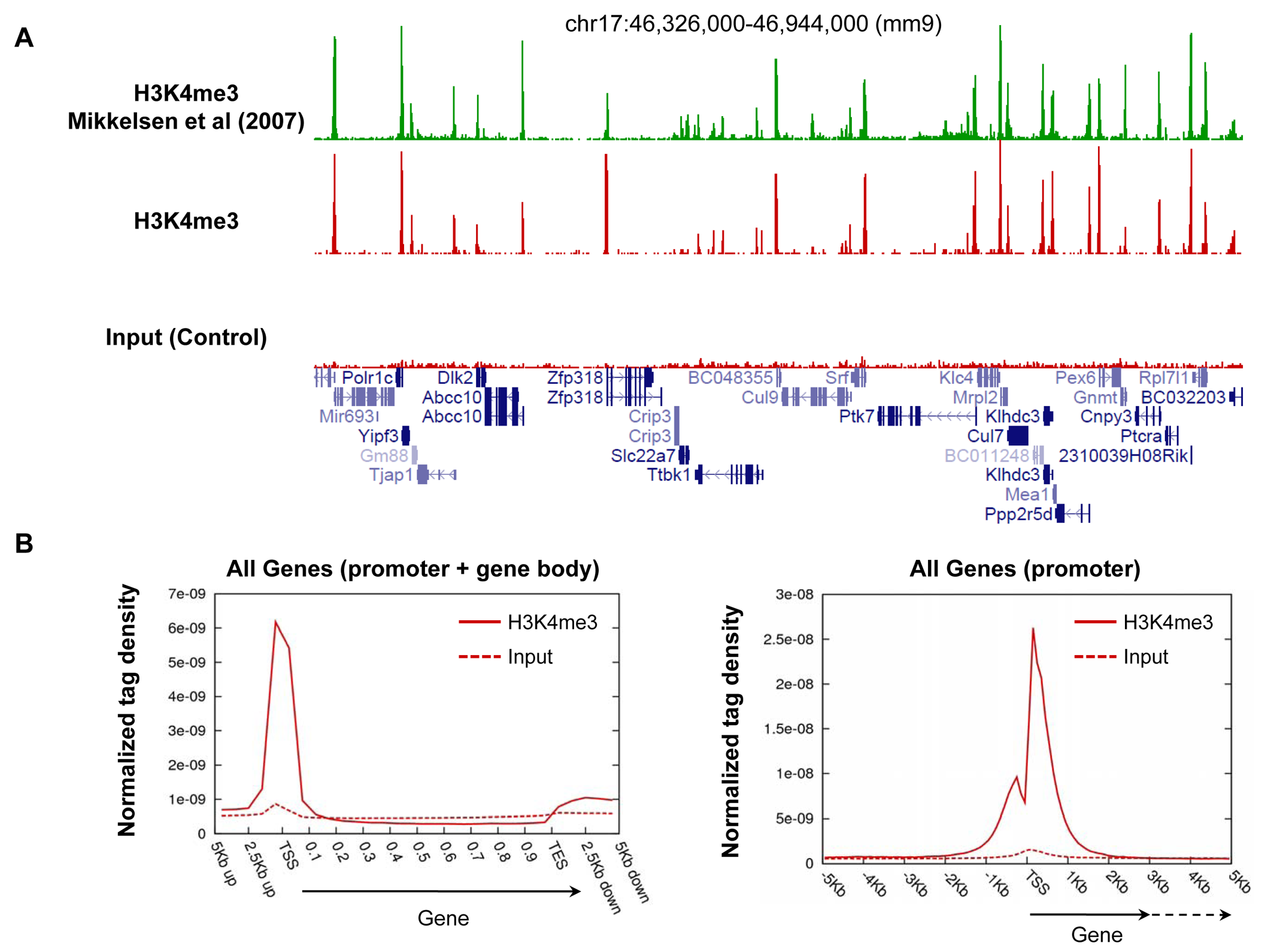

Supplement: Figure S1 — Data quality of genome-wide H3K4me3 ChIP-Seq from mESCs. (A) UCSC genome browser images of H3K4me3 profiles (top two tracks) at a randomly selected ∼600 Kb region on chromosome 17 in wild-type mESCs. The top track shows H3K4me3 data from a previously published report (17). The middle and bottom track shows the data (H3K4me3 and input, respectively) generated for this study. Genes within the locus are shown at the bottom. (B) Normalized average tag density across a gene unit (left) and 10 Kb surrounding the transcription start site (TSS) (right) is shown. All genes in the mouse genome were used to calculate the average tag density. (TIF) [file pone.0037952.s001.tif]

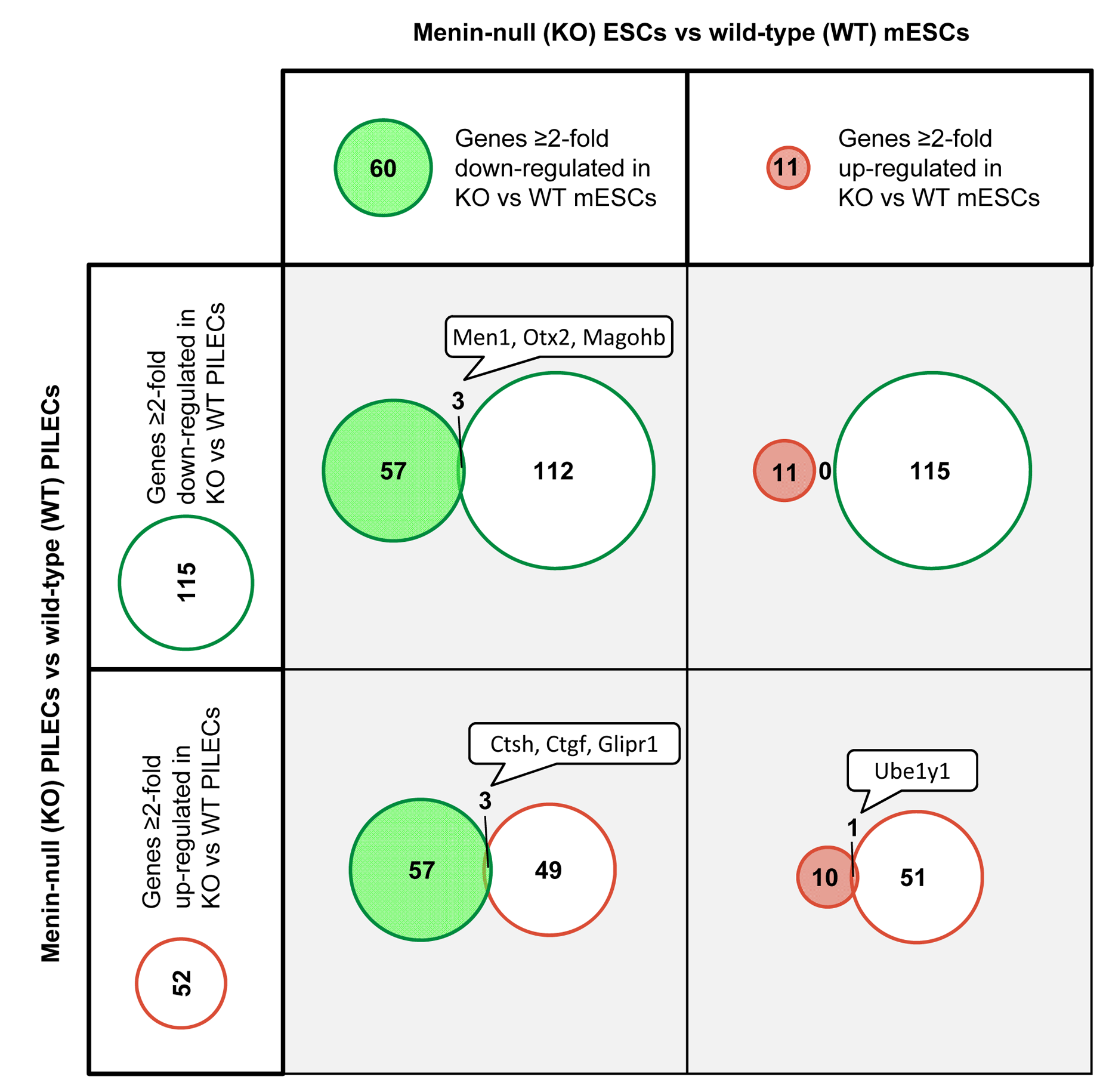

Supplement: Figure S2 — Differentially expressed genes in menin-null cells. Number of genes that were at least 2-fold differentially expressed (p<0.005) in menin-null vs wild-type (WT) mESCs and menin-null vs WT pancreatic islet-like endocrine cells (PILECs) are shown. Overlaps between the differentially expressed subsets are represented as venn diagrams. (TIF) [file pone.0037952.s002.tif]
